# Transcriptomic Characterization of a Synergistic Genetic Interaction during Carpel Margin Meristem Development in *Arabidopsis thaliana*

**DOI:** 10.1371/journal.pone.0026231

**Published:** 2011-10-21

**Authors:** April N. Wynn, Elizabeth E. Rueschhoff, Robert G. Franks

**Affiliations:** 1 Department of Genetics, North Carolina State University, Raleigh, North Carolina, United States of America; 2 Department of Biology, Indiana University Southeast, New Albany, Indiana, United States of America; Kyushu Institute of Technology, Japan

## Abstract

In flowering plants the gynoecium is the female reproductive structure. In *Arabidopsis thaliana* ovules initiate within the developing gynoecium from meristematic tissue located along the margins of the floral carpels. When fertilized the ovules will develop into seeds. *SEUSS* (*SEU*) and *AINTEGUMENTA* (*ANT*) encode transcriptional regulators that are critical for the proper formation of ovules from the carpel margin meristem (CMM). The synergistic loss of ovule initiation observed in the *seu ant* double mutant suggests that *SEU* and *ANT* share overlapping functions during CMM development. However the molecular mechanism underlying this synergistic interaction is unknown. Using the ATH1 transcriptomics platform we identified transcripts that were differentially expressed in *seu ant* double mutant relative to wild type and single mutant gynoecia. In particular we sought to identify transcripts whose expression was dependent on the coordinated activities of the *SEU* and *ANT* gene products. Our analysis identifies a diverse set of transcripts that display altered expression in the *seu ant* double mutant tissues. The analysis of overrepresented Gene Ontology classifications suggests a preponderance of transcriptional regulators including multiple members of the *REPRODUCTIVE MERISTEMS* (*REM)* and *GROWTH-REGULATING FACTOR* (*GRF)* families are mis-regulated in the *seu ant* gynoecia. Our *in situ* hybridization analyses indicate that many of these genes are preferentially expressed within the developing CMM. This study is the first step toward a detailed description of the transcriptional regulatory hierarchies that control the development of the CMM and ovule initiation. Understanding the regulatory hierarchy controlled by *SEU* and *ANT* will clarify the molecular mechanism of the functional redundancy of these two genes and illuminate the developmental and molecular events required for CMM development and ovule initiation.

## Introduction

In both gymnosperms and angiosperms, ovules are critical for reproductive competence. Ovules contain the female gametophyte and thus the egg cell. Additionally, upon fertilization the ovules develop into the seeds that nurture and protect the developing embryos. In *Arabidopsis thaliana*, two rows of ovules develop from a ridge of meristematic tissue on the inner surface of the seed pod or gynoecium. Within the developing ovule primordia, much is known about molecular patterning events along the proximal to distal axis and the mechanisms of integument development [1], [2], [3]. Also dramatic progress has been made with respect to understanding the subsequent development of the female gametophyte within the maturing ovule [4], [5], [6]. However, considerably less is known about the earliest steps in ovule development: the mechanisms of ovule initiation, and in the establishment and maintenance of the meristematic tissues of the carpel margin meristem (CMM) that generate the ovule primordia.

**Figure 1:**
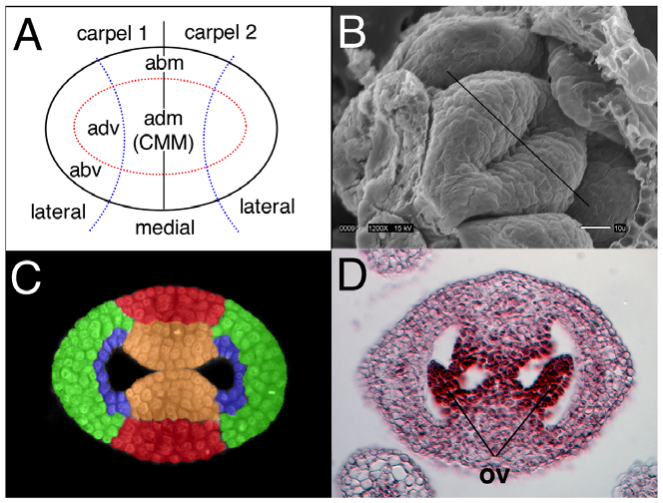
Spatial domains of the developing Arabidopsis gynoecium. A) Diagrammatic representation of the Arabidopsis gynoecial primordia at stage 6. Blue dotted arcs separate the lateral domains from the medial domain. The medial domain represents the fused margins of the two component carpels. The red dotted oval separates abaxial (outer) positions from adaxial (inner) positions. adm - adaxial margin; abm - abaxial margin; adv - adaxial valve; abv - abaxial valve, CMM - carpel margin meristem. B) Scanning electron micrograph of stage 6 gynoecial primordium. Medial plane is marked with a black line. C) False colored confocal cross section of a stage 8 gynoecium. Gynoecial domains have been colored with approximation. orange - carpel margin meristem/medial ridge; red - abaxial margin/replum; blue - adaxial valve; green - abaxial valve. D) Histological cross section of a stage 11 Arabidopsis gynoecium. Ovules (ov) are indicated.

Gynoecial development in Arabidopsis initiates at stage 6 of floral development (floral stages according to Smyth; [7]). The gynoecial primordium is first morphologically recognizable as a dome or mound of cells, oval in cross section, that forms from the cells of the central most portion of floral meristem (i.e. floral whorl 4). During stage 6 the different spatial domains of the gynoecial tube are already discernable based on the differential expression of genes within the medial portion of the gynoecium versus the lateral domains, as well as along the inner to outer (adaxial to abaxial) axis [8], [9], [10] (Fig. 1A). During floral stages 6 and 7 the proliferation of cells along the perimeter of the gynoecial dome leads to the formation of a tube-shaped structure (Fig. 1B).

The single gynoecium primordium likely represents a composite of two congenitally-fused carpel organs in a phylogenetic sense (Fig. 1A) [8], [11]. In this scenario, the medial portions of the gynoecium represent the fused margins of the two component carpels. The adaxial portions of the medial/marginal domain maintain meristematic potential throughout the elongation of the gynoecial tube and these regions have been termed carpel margin meristems (CMMs) [12], [13], [14]. Each Arabidopsis gynoecium contains two CMMs that are positioned within the adaxial portions of the medial domain of the gynoecium. During floral stages 7 and 8 the CMM takes the shape of a ridge of tissue (the medial ridge) that extends along the apical basal extent of the gynoecial tube (Fig. 1C). During mid to late stage 8 each CMM gives rise to two rows of ovule primordia from the peripheral portions of the meristematic ridge (Fig. 1D). Later, the CMM also gives rise to the gynoecial septum and transmitting tract and likely generates portions of the stigmatic and stylar tissues. A variety of data suggests that the proper specification of adaxial and medial/marginal positional identities are important for the development of the CMM and subsequent ovule initiation [12], [15].

### *SEU* and *ANT* act synergistically during CMM development

A number of genes have been suggested to play a role in the maintenance of meristematic potential in the CMM and for the subsequent initiation of ovule primordia from the flanks of the CMM. While no single mutant has been reported to strongly disrupt ovule initiation, several higher order mutant combinations have been reported to disrupt the initiation of ovule primordia from the CMM [12], [13], [14], [16], [17], [18], [19], [20]. The *seuss aintegumenta* double mutant is one such genetic mutant combination [12]. The number of ovule primordia in the *seuss* (*seu*) single mutant is nearly wild type while the *aintegumenta* (*ant*) mutant conditions the loss of about 50% of the ovule primordia. Together the loss of both the *SEU* and *ANT* activities in the *seu ant* double results in the complete loss of ovule initiation, indicating a synergistic genetic interaction and suggesting a degree of overlapping function for *SEU* and *ANT* during CMM development.

*SEU* and *ANT* both encode transcriptional regulators [21], [22], [23]. *ANT* encodes an *AP2*-type DNA binding transcription factor that is expressed in all lateral organ primordia (leaves, floral organs, ovules) [22], [23]. Within the context of early gynoecial development, *ANT* is expressed throughout the stage 6 gynoecial mound with a higher level of expression within the adaxial core (central portions) [12], [23]. At late stage 7 and early stage 8 expression of *ANT* is strong in the ovule anlagen and early ovule primordia as they arise. *ANT* activity during primordium development supports organ growth by maintaining the developmental period during which cell growth and cell divisions occur [24], [25]. *ANT* has also been shown to contribute to proper specification of floral organ identity and polarity specification [18], [26], [27]. While direct targets of *ANT* regulation have not yet been published, *PHB* and cyclinD3 have been shown genetically to be downstream of *ANT* regulation [12], [18], [24], [28] further supporting a role for *ANT* in organ polarity specification and regulation of cellular proliferation and/or organ growth.

*SEU* encodes a transcriptional adaptor protein that is expressed widely throughout the plant [12], [21]. *SEU* does not have a specific DNA binding activity but rather complexes with sequence specific DNA binding proteins in order to exert its effects on transcriptional regulation [29], [30]. The best-characterized functional role for SEU is in the repression of *AGAMOUS* (*AG*) expression during floral organ identity specification [21]. In this context SEU interacts with pairs of MADS-domain containing DNA transcription factors and recruits the transcriptional repressor LEUNIG to the second intron of the AG gene [29], [30], [31]. The binding of this complex is thought to bring about repression of *AG* transcription through the recruitment of histone deacetylase proteins [30], [32].

### Adaxial fate specification is compromised in the *seu ant* double mutant

A variety of experimental data suggest that the disruption of CMM development observed in the *seu ant* mutant is not conditioned simply by a de-repression of AG, but rather that *SEU* and *ANT* function to maintain or specify adaxial fate in the gynoecium and that this fate specification is critical for proper CMM development [12], [16], [19]. These studies demonstrated that expression levels of *PHABULOSA* (*PHB*) and *REVOLUTA* (*REV*) are reduced in the adaxial core of the stage 6 gynoecium in *seu ant* mutant plants. *PHB* and *REV* encode transcriptional regulators of the Homeodomain Leucine Zipper Class III type (HDZip-III) that are known to play a key role in the specification of adaxial identity in lateral organs [33], [34], [35], [36], [37], [38], [39], [40]. These genetic studies, however, were not able to determine if the effect of the loss of *SEU* and *ANT* activity on HDZip-III expression was due to a direct or indirect regulation of their expression or accumulation. Additionally the defects in ovule and CMM development observed in the *seu ant* double mutant were not rescued when *PHB* activity was replaced, suggesting that either that *PHB* could not substitute for the other HDZip-III family members or that gene functions in addition to HDZip-IIIs are required downstream of *SEU* and *ANT* for CMM development [12]. Synergistic disruptions of gynoecial and CMM development observed in the *ant rev* double mutant, but not in *ant phb* double mutant support the idea of a functional differentiation between the *PHB* and *REV* activities within the CMM [15]. The analysis of higher order mutants of the HDZip-III family members also suggests a diversification of functional roles within this gene family [40]. However these data do not exclude the possibility that there are a large number of additional gene regulation events critical for CMM development downstream of *SEU* and *ANT* that remain to be elucidated.

### Genetic analyses reveal a complex and highly redundant mechanism supporting CMM development

Although no single mutant has been identified that eliminates CMM development or ovule initiation, a number of double mutant or higher order mutant combinations condition a severe disruption of the CMM and CMM-derived tissues (e.g. ovules) [12], [16], [18], [19], [20], [27]. These data suggest that one or more redundant genetic programs support the development of the CMM. A portion of this resiliency is likely supported by the action of multiple members of structurally related genes families. Both *SEU* and *ANT* are members of gene families whose members have been shown to share redundant function [16], [27]. With respect to the CMM, the *SEUSS-LIKE* genes, *SLK1*, and *SLK2* genetically enhance the *ant* mutant phenotype with respect to ovule initiation defects [16]. Similarly the *ANT-LIKE* family member, *AIL6,* shares a critical redundant function with *ANT* as the *ant ail6* double mutants flowers display reduced medial domain development and initiate very few ovule primordia [27]. Other mutant combinations indicate instances of molecularly dissimilar molecules sharing overlapping functions during floral and CMM development. A redundant function shared between *ANT* and the *YABBY* family members *YAB1* and *YAB3* is suggested by the synergistic disruption of ovule initiation observed in the *ant yab1* and *ant yab1yab3* mutants [18]. Analysis of *ant shatterproof1 (shp1) shatterproof2 (shp2) crabs claw (crc)* mutants implicates the *SHP* MADS domain transcription factors in CMM development. These studies together highlight an important role for *ANT* function during CMM development and ovule initiation as well as reveal a high degree of functional redundancy within this tissue.

### A high degree of redundancy hinders genetic approaches to the study of the CMM

A number of key regulators of CMM development may be difficult to recover with standard forward genetic approaches due to a high degree of redundancy. Identifying genes that have specific patterns of spatial and temporal expression in the CMM would generate a set of candidate genes that could then be analyzed by reverse genetic approaches. In this paper we employ a transcriptomic profiling approach to identify sets of genes that are differentially expressed in the developing carpels of the *seu ant* double mutant. In particular we sought to identify transcripts whose expression was dependent on the coordinated activities of *SEU* and *ANT* gene products. We hoped to both identify novel regulators of CMM development and to examine the molecular mechanism of the functional redundancy of *SEU* and *ANT* during CMM development. Our analysis identified a diverse set of transcripts that display altered expression in the *seu ant* double mutant tissues. Our *in situ* hybridization analyses indicate that many of these genes are preferentially expressed within the developing CMM. The analysis of overrepresented Gene Ontology classifications suggests a preponderance of transcriptional regulators including multiple members of the *REPRODUCTIVE MERISTEMS* (*REM)* and *GROWTH-REGULATING FACTOR* (*GRF)* families of transcriptional regulators are mis-regulated in the *seu ant* gynoecia. This study is the first step toward a detailed description of the transcriptional regulatory hierarchies that control the development of the CMM and ovule initiation.

## Results

### Transcriptomic analysis reveals putative targets of *SEU* and *ANT* regulation important for CMM development

In an effort to identify novel regulators of CMM development and ovule initiation we identified genes that are preferentially expressed within the CMM within the context of the gynoecium. Additionally we endeavored to prioritize genes whose expression is synergistically disrupted in the *seu ant* double mutant relative to either single mutant. We isolated RNA from staged (floral stages 8 through 10) and hand-dissected gynoecia to limit the developmental window of the sample to the period just before and then during ovule primordia initiation, the earliest steps of ovule development. This differentiates our work from that of the Gasser and Colombo groups that have focused on later ovule developmental stages when identifying ovule-specific transcripts [2], [41].

We utilized the Arabidopsis ATH1 Gene Chip (Affymetrix) to compare transcript levels between four different genotypes (Col-0, *seu-3, ant-1,* and *seu-3 ant-1* double mutant). We first analyzed mRNA accumulation in each single mutant relative to the Col-0 wild type gynoecial samples. To identify transcripts whose steady-state levels were altered in the single mutants relative to wild type, we utilized a 1-way ANOVA and identified probe sets (transcripts) that displayed a statistically significant difference in accumulation by the genotype term. This analysis identified 120 under-expressed and 200 over-expressed transcripts in the *seu* single mutant and 219 under-expressed and 241 over-expressed transcripts in the *ant* single mutant (Tables S1, S2, S3, S4) Throughout this manuscript we refer to transcripts that display a differential steady state level of accumulation in a given sample as differentially “expressed” with the caveat that we are measuring steady state levels and cannot differentiate transcriptional from post-transcriptional effects on RNA accumulation with these approaches.

Over-represented GO categories for the genes displaying reduced expression in *seu* are reported in Table S5 and include “sequence specific DNA-binding transcription factor activity” (GO:0003700) and “leaf development” (GO:0048366). For genes that are over-expressed in the *seu* single mutant the over represented GO categories are reported in Table S6. Over represented GO categories for the genes under-expressed in *ant single* are reported in Table S7 and include “sequence specific DNA-binding transcription factor activity” (GO:0003700) and “flower development” (GO:0009908). Over represented GO categories for the genes over-expressed in *ant* single are reported in Table S8.

As the *seu ant* double mutant fails to initiate ovule primordia, we reasoned that genes critical for the earliest steps of ovule initiation would display reduced expression in the *seu ant* double mutant gynoecia, relative to either single mutant or the wild-type tissues. To identify transcripts that are differentially expressed in the *seu ant* double mutant relative to the other genotypes, we utilized two statistical approaches. For Approach I we used a 1-way ANOVA to identify probe sets for which the mean expression level was significantly different in the double mutant relative to the overall expression mean: 210 transcripts displayed reduced accumulation (Table S9) and 128 displayed elevated accumulation in the double mutant using this analysis approach (Table S10). In the set of genes with reduced accumulation in the *seu ant* double mutant statistically enriched GO categories included “transcription factor activity”, “ad/abaxial polarity specification”, “flower development”, and “transmembrane receptor protein kinase activity” (Table S11). The GO terms that were significantly enriched in the gene set with elevated accumulation are presented in Table S12.

We focused our attention on the genes that displayed **reduced** expression within the *seu ant* double mutant because: 1) over-represented GO terms suggest a role for this gene set in transcriptional regulation and relevant developmental processes, and 2) the reduced accumulation of these transcripts in the *seu ant* double mutant suggests that they may be preferentially expressed in the CMM in the wild-type gynoecium and, thus, are candidates for novel regulators of CMM development.

### Analytical Approach II yields 31 high-priority putative CMM regulators

To further identify genes exhibiting reduced expression in the *seu ant* double mutant we used a second analytical approach (Approach II) comprised of two steps. We first selected probe sets for which the mean expression was significantly lower in the *seu* or *ant* single mutant relative to wild type (The union of the genes sets reported in Tables S3 and S4). As the *seu-3* and *ant-1* single mutants display very minor morphological disruptions in stage 8 and 9 gynoecia, we reasoned that transcripts displaying reduced accumulation in the single mutants would not simply reflect a morphological loss of CMM tissue in these samples, but might be more likely to reflect a reduction in the level of transcription of a given gene in the mutant. We then applied a second selection criterion such that we additionally required that the transcript abundance in *seu-3 ant-1* double mutant gynoecia be lower than an expected value that was estimated via an additive model using the data from each single mutant. This was done with the JMP Genomics estimate builder with a significance cutoff of alpha <0.05. By using these selection criteria, we hoped to enrich for genes that were synergistically reduced in expression in the *seu-3 ant-1* double mutant and that might uncover the molecular basis of the synergistic phenotypic enhancement in the *seu ant* double mutant.

Approach II yielded just 31 candidate genes (Table 1). Hereafter referred to collectively as “Approach II candidate genes”. The majority (55%) of the Approach II candidate genes encode transcriptional regulators. Several observations suggest that many of these candidates are preferentially expressed in the developing carpel margin and are likely important regulators of CMM development that are downstream of *SEU* and *ANT* regulation. Firstly, twenty-eight of the thirty-one Approach II candidate genes were also found within the set of 210 genes showing significantly reduced accumulation in the double mutant as identified by Approach I. Secondly, 11 of the 31 genes have been previously shown to be expressed preferentially in the CMM or in CMM-derived tissues (e.g. ovules). These include *AT1G02800* (*ATCEL2*), *AT3G55560* (*AGF2*), *AT5g57720* (*REM15*) *AT2g46870* (*NGA1*), *AT1G68640* (*PAN*), *AT2G34710* (*PHB*), *AT4G37750* (*ANT*), *AT1G70560* (*TAA1*), *AT5G18000* (*VDD*), *AT3G17010* (*REM22*), and *AT4G31610* (*REM34* - previously *AtREM1*) [2], [12], [15], [22], [23], [40], [41], [42], [43], [44], [45], [46], [47], [48]. The expression levels of two of these (*PHB* and *TAA1*) has been previously shown to be reduced in *seu*, *ant* or *seu ant* mutant gynoecia [12], [15], [16]. Interestingly even though *SEU* and *ANT* have been implicated in the repression of *AG* in perianth organs, the levels of *AG* expression were not significantly different from wild type in the *seu* or *ant* single mutant gynoecial RNA samples (Table 3).

**Table 1:** Approach II Candidate Genes.

AGI	Gene Title	GO Category Transcriptional Regulator	B3 Family member	wt log2 Lsmean	*ant* log2 Lsmean	*seu* log2 Lsmean	*seu ant* log2 Lsmean	−log(10) P-value*
At2g46870	*NGATHA1-* B3 domain	*+*	+	8.42	8.52	7.85	7.17	5.06
At3g17010	*REM22* - B3 domain	*+*	+	10.03	9.80	8.60	7.37	2.98
At3g53310	*REM16* - B3 domain	*+*	+	11.09	11.00	10.50	9.93	1.58
At5g18000	*REM20, VERDANDI -* B3 domain	*+*	+	8.55	7.72	8.54	7.02	3.51
At5g57720	*REM15 -* B3 domain	*+*	+	9.45	9.48	8.97	8.45	2.01
At3g19184	*REM1* - B3 domain	*+*	+	8.59	7.77	8.37	7.08	1.65
At4g31610	*REM34 -*B3 domain	*+*	+	10.18	10.12	9.53	8.98	1.12
At4g24150	*AtGRF8, transcriptional regulator*	*+*		9.56	9.03	9.35	8.24	1.78
At1g31310	myb-like domain	+		9.64	9.71	9.22	8.86	1.84
At1g51950	*IAA18,* transcription factor	*+*		9.56	9.18	9.37	8.33	4.17
At1g68640	*PERIANTHIA,* DNA binding	*+*		9.82	9.04	9.31	8.09	1.22
At2g34710	*PHABULOSA*, transcription factor	*+*		9.49	9.21	8.95	8.24	2.90
At3g13960	*AtGRF5,* transcriptional regulator	*+*		9.21	9.30	8.25	7.84	1.61
At3g55560	*AGF2,* DNA-binding protein	*+*		8.47	8.00	8.22	7.26	2.18
At4g00180	*YABBY3*, transcription factor	*+*		9.27	8.38	8.64	7.05	2.50
At4g37750	*AINTEGUMENTA,* DNA binding	*+*		10.80	9.65	10.69	8.91	2.18
At5g61850	*LEAFY,* transcription factor	*+*		9.76	8.96	8.90	7.41	1.58
At1g02800	*ATCEL2,* Cellulase hydrolase			11.93	11.47	11.18	9.95	1.90
At1g68780	leucine-rich repeat family protein			8.41	8.26	7.74	6.87	5.94
At2g27880	*AGO5,* argonaute protein			10.19	9.65	9.98	8.94	2.01
At3g21560	*UGT84A2;* UDP-glycosyltransferase			9.73	9.71	8.87	8.37	1.38
At1g01110	*IQD18,* calmoduin binding			8.85	8.78	8.30	7.63	4.87
At1g03710	cystatin-related			10.19	10.16	9.78	8.81	4.06
At1g03720	cathepsin-related			9.00	8.16	8.41	7.05	2.34
At1g70560	*TAA1* - auxin synthesis			9.61	9.07	9.07	7.62	4.90
At1g73590	*PIN1,* auxin transporter			10.42	9.99	10.04	8.70	4.02
At2g21050	*LAX2* - auxin influx carrier			9.95	9.57	10.23	8.76	6.11
At4g25240	*SKS1* (*SKU5 SIMILAR 1*)			9.77	10.09	9.16	8.75	3.63
At5g07280	*EXCESS MICROSPOROCYTES1* kinase			10.47	10.54	10.03	9.42	4.56
At5g17080	cathepsin-related			9.42	8.56	9.53	6.81	6.34
At5g48900	pectate lyase family protein			8.68	8.39	8.22	7.50	2.01

*–log(10) of P-value returned by JMP Genomics estimate builder.

### qRT verification of candidates

From this set of 31 genes that displayed reduced expression in the *seu ant* double mutant we have confirmed by qRT PCR nine out of ten genes tested (Table 2). We also confirmed an additional 7 of 7 genes that displayed increased expression in the *seu ant* double mutant (Table 3).

**Table 2:** qRT PCR verification of candidates under-expressed in the *seu ant* double mutant.

		wild type			*seu* mutants			*ant* mutants			*seu ant* double mutants		
Gene ID	Gene Name	expression mean*	standard error mean	Tukey HSD level§	expression mean*	standard error mean	Tukey HSD level§	expression mean*	standard error mean	Tukey HSD level§	expression mean*	standard error mean	Tukey HSD level§
At4g31610	*REM34*	0.43	0.03	A	0.25	0.04	B	0.33	0.01	AB	0.12	0.02	C
At2g46870	*NGA1*	0.11	0.01	A	0.04	0.003	C	0.09	0.004	B	0.01	0.001	D
At1g68640	*PAN*	0.16	0.02	A	0.08	0.003	B	0.06	0.005	BC	0.02	0.005	C
At1g31310	myb-domain	n.d.	-	-	n.d.	-	-	n.d.	-	-	n.d.	-	-
AT3G61970	*NGA2*	0.03	0.002	A	0.01	0.003	C	0.02	0.002	B	0.001	0.0002	C
At3g55560	*AGF2*	0.07	0.01	A	0.05	0.005	B	0.03	0.004	BC	0.01	0.002	C
At3g53310	*REM16*	15.78	0.91	A	7.35	0.41	C	12.25	0.60	B	4.15	0.67	D
At3g21560	*UGT84A2*	0.55	0.03	A	0.22	0.04	B	0.41	0.04	A	0.11	0.02	B
At5G18000	*VDD*	0.12	0.01	A	0.12	0.02	A	0.04	0.01	B	0.01	0.001	B
At1g68780	LRR type	0.11	0.01	A	0.04	0.00	C	0.07	0.01	B	0.01	0.001	D

*mean normalized expression [normalized to *ADENOSINE PHOSPHORIBOSYL TRANSFERASE1* (*At1g27450*)].

§Tukey honest statistical difference test level.

n.d. not detected.

**Table 3:** qRT PCR verification of candidates over-expressed in the *seu ant* double mutant

		wild type			*seu* mutants			*ant* mutants		*seu ant* double mutants			
Gene ID	Gene Name	expression mean*	standard error mean	Tukey HSD level§	expression mean*	standard error mean	Tukey HSD level§	expression mean*	standard error mean	Tukey HSD level§	expression mean*	standard error mean	Tukey HSD level§
At1g20450	*ERD10*	0.03	0.01	C	0.09	0.03	B	0.03	0.002	C	0.16	0.02	A
At2g15970	*COR413*	0.05	0.01	C	0.16	0.02	B	0.03	0.01	C	0.30	0.04	A
At1g26960	*ATHB23*	0.03	0.004	C	0.06	0.004	B	0.03	0.001	C	0.11	0.01	A
At2g33380	*RD20*	0.04	0.01	BC	0.08	0.005	B	0.02	0.003	C	0.15	0.03	A
At1g05850	*POM1*	0.40	0.04	B	0.42	0.01	B	0.35	0.03	B	0.66	0.07	A
At1g69780	*ATHB13*	0.23	0.04	BC	0.19	0.02	C	0.29	0.02	B	0.45	0.02	A
At3g11090	*LBD21*	0.03	0.004	B	0.04	0.003	B	0.02	0.003	B	0.08	0.01	A
		**wild type**			***seu*** ** mutants**			***ant*** ** mutants**		***seu ant*** ** double mutants**			
At4g18960^#^	*AG*	19.23	2.36	A	20.21	2.01	A	15.82	1.06	A	22.94	3.23	A

*mean normalized expression [normalized to *ADENOSINE PHOSPHORIBOSYL TRANSFERASE1* (*At1g27450*)].

§Tukey honest statistical difference test level.

# Expression level of *AG* was not statistically different across the four genotypes in data from the ATH1 arrays, nor as estimated with qRT PCR.

### *REM* family and *AtGRF* family transcriptional regulators are significantly over-represented in Approach II candidates

Surprisingly, seven out of the 31 Approach II candidate genes were members of the *B3* superfamily of transcription factors (Table 1) [49], [50]. This enrichment for genes encoding *B3* transcription factors within our sample is highly unlikely to have occurred by chance alone (p = 2.5×10^−10^ by hypergeometric probability test). The Arabidopsis *B3* superfamily consists of 118 genes all of which encode proteins containing one or more *B3*-type DNA binding domains. The *B3* superfamily is comprised of four sub-families: *REM (REPRODUCTIVE MERISTEM); LAV (LEAFY COTYLEDON2[LEC2]-ABSCISIC ACID INSENSITIVE3 [ABI3]-VAL); ARF(AUXIN RESPONSE FACTOR);* and *RAV(RELATED TO ABI3* and *VP1)*. Six of the seven *B3* regulators that were identified in our transcriptomics approach are from the *REM* subfamily for which there is little functional data. Four of these genes, *AT4G31610* (*REM34/AtREM1*), *AT5G18000* (*VDD*), *At5G57720* (*REM15*) and *AT3G17010* (*REM22*), have been previously reported to display CMM-enriched expression [41], [42], [43], [47], [51].

The *GRF* family of genes is also overrepresented in the list of Approach II candidates (p = 6.9×10^−5^ by hypergeometric probability test). Members of the *GRF* family in *Arabidopsis* have been shown to regulate growth and development of leaves, cotyledons and floral organs [52], [53], [54]. Over expression of members of this gene family result in larger and wider leaves while *grf5* single mutants and *grf1,2,3* triple mutants display narrower leaves, suggesting a role in the regulation of cell proliferation within the medial to lateral axis of the leaf.

### Expression of several *REM* family genes marks the gynoecial medial domain

We used *in situ* hybridization to further characterize the temporal and spatial expression patterns of a number of the Approach II candidates during early gynoecial development. Although for several of these genes expression data from *in situ* hybridization experiments were previously published, these data did not examine the expression pattern of these genes in detail during gynoecial development. We specifically focused on the developing gynoecium and examined cross sections to determine the expression patterns within the medial versus lateral gynoecial domains. In some cases we also examined the expression in *seu, ant*, and *seu ant* double mutant tissues. Our results indicate that all six *REM* family members identified with Approach II analysis are expressed preferentially within the medial gynoecial domain with varying developmental profiles.

**Figure 2:**
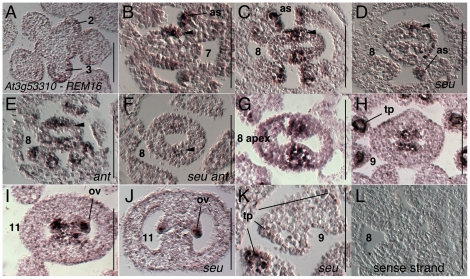
Results of *in situ* hybridization with *At3g53310* (*REM16*) antisense probe. Results of *in situ* hybridization with *At3g53310* (*REM16*) antisense probe (A–K) or with sense strand control probe (L). Numbers indicate floral stages. All panels show transverse (cross sectional) tissue orientation. Arrowheads indicate medial domain expression; ov - ovule; as, archesporial precursors; tp, tapetum. All scale bars are 100 microns. All panels are Col-0 wild type tissue unless otherwise indicated.

*At3G53310* (*REM16*) was previously reported in stamen primordia at stage 4 and carpel primordia at stage 6 [43]. We detected expression of *At3G53310* (*REM16*) weakly in the stage 1–4 floral primordia, chiefly in L1 layer and in peripheral portions of the floral meristem that will give rise to the sepal primordia (Fig. 2A). Expression is also detected in stamen and petal primordia as they arise at stage 4 and 5 (data not shown). Expression in the gynoecium is difficult to detect before early stage 7 when expression is observed in the abaxial portions of the medial gynoecial domain (Fig. 2B). Expression during stage 8 is seen in the medial domains and begins to be detected in both adaxial and abaxial portions. However expression is not observed in the L1 layer (Fig. 2C and G). The stage 8 medial domain expression appeared reduced in the *seu* single and the *seu ant* double mutant relative to wild type (Figs. 2D, E, F). In wild type tissue expression continues to be detected in the ovule primordia throughout stages 9 through 11 and is confined to subepidermal cell layers (Fig. 2I). During stage 7 and 8 expression in the stamens is detected mostly in the subepidermal cells from which archesporial and tapetal cells are derived (Fig. 2 C, H). During stage 9 expression is most strongly detected in the tapetal cells (Fig. 2 H). Expression within the stamen primordia also appeared to be reduced in the *seu* mutant tissues (Fig. 2D, K). Hybridizations with sense strand probes gave very little background staining (Fig. 2L).

**Figure 3:**
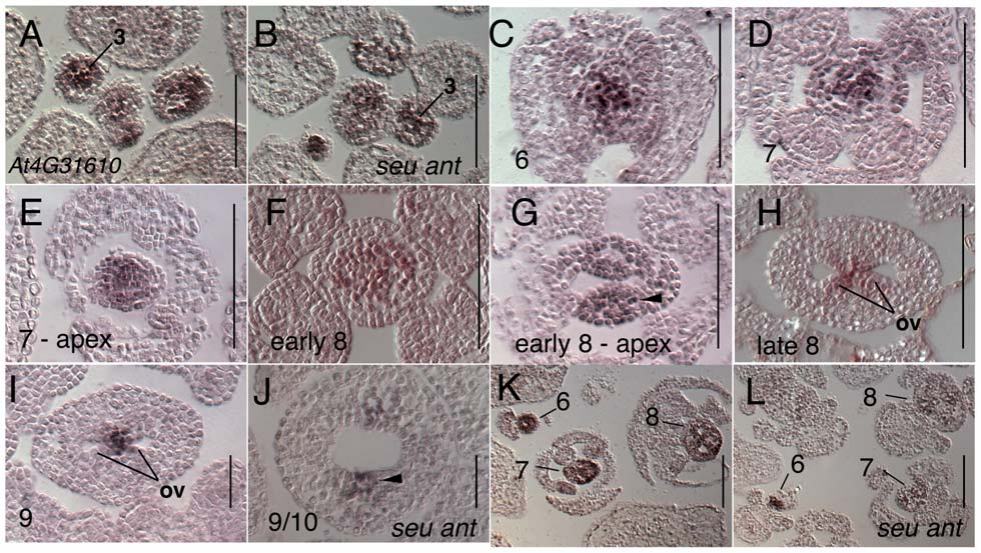
Results of *in situ* hybridization with *At4G31610* (*REM34/AtREM1)* antisense probe. Numbers indicate floral stages. All panels show transverse (cross sectional) tissue orientation. Arrowheads indicate medial domain expression; ov - ovule. All scale bars are 100 microns. All panels are Col-0 wild type tissue unless otherwise indicated.

Expression of *At4G31610* (*REM34/AtREM1*) in the inflorescence meristem and in floral stages 2–5 has been previously reported [42]. Franco-Zorrilla *et al.* also report expression of *AT4G31610* (*REM34/AtREM1*) is confined to gynoecial primordium from stage 6 onward and later expressed in the medial ridge, septum, style and stigma [42]. Our analysis of gynoecial expression patterns reveals that *At4G31610* (*REM34/AtREM1*) is expressed in the adaxial core of the stage 6 and stage 7 gynoecial primordia (Fig. 3C and D). During stage 7 and 8 expression is strongest at the apex of the gynoecium in the medial domain (Fig. 3E, G). During late stage 8 expression is detected in the ovule anlagen (Fig. 3H) while by stage 9 expression appears to be restricted to the cells that lie between the ovule primordia and are likely to be the progenitors of the gynoecial septum. Expression in the *seu ant* double mutant tissue appeared reduced in the gynoecial primordia during stages 6–10 but was similar to wild type during stage 2–3 (Fig. 3 A, B, J and L).

**Figure 4:**
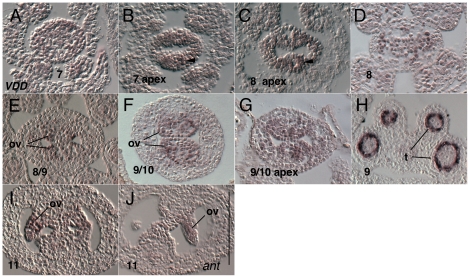
Results of *in situ* hybridization with *AT5G18000* (*VDD*) antisense probe. Numbers indicate floral stages. All panels show transverse (cross sectional) tissue orientation. Arrowheads indicate medial domain expression; ov - ovule; t- tapetal cells. Scale bar in J represents 100 microns for all panels. All panels are Col-0 wild type tissue unless otherwise indicated.

Expression of *AT5G18000* (*VDD*) was previously reported in inflorescence and floral meristems and in ovules, as well as within the developing female gametophyte [41]. Within the gynoecium we first observe expression of *VDD* at stage 7 when it is detected weakly throughout the primordium (Fig. 4A). Expression was stronger at the medial portions of the apex of the stage 7 and 8 gynoecial primordia relative to more basal positions (Fig. 4B, C). Expression continues in the apical medial domain into stage 10 (Fig. 4G). Expression is also detected in ovule primordia as they arise at stage 8 and continues throughout ovule development (Fig. 4E and data not shown) [41]. Expression in ovule primordia at stage 11 was strongest in the chalazal portions of the ovule. Expression in the *ant* single mutant tissue at this stage appeared reduced, suggesting that *ANT* may regulate the expression of *VDD* in the chalazal portions of the ovule. Strong expression was also detected in tapetal cells of the anther at stage 9 (Fig. 4H).

**Figure 5:**
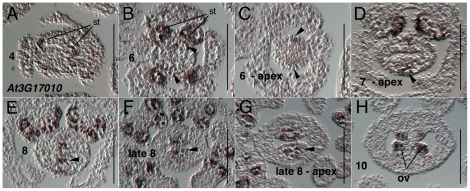
Results of *in situ* hybridization with *AT3G17010* (*REM22*) antisense probe. Numbers indicate floral stages. All panels show transverse (cross sectional) tissue orientation. Arrowheads indicate medial domain expression; ov - ovule; st- stamen primordia. Scale bars in all panels are 100 microns. All panels are Col-0 wild type tissue. Oblique section in panel B skews apparent location of medial domain slightly.

Expression of *At3G17010 (REM22)* in stamen and carpel primordia has been previously reported [47], [51]. We first detected expression of *AT3G17010* starting at late stage 4 or early stage 5 in the stamen primordia as they arise (Fig. 5A). Within the gynoecium expression can be detected preferentially in the medial domain as early as stage 6 (Fig. 5B, C). *AT3G17010* continues to be preferentially expressed subepidermally within the medial domain through stage 8 (Fig. 5E, F) and is strongly detected in the medial domain at apical positions of the stage 8 gynoecium (Fig 5G). Expression is detected in the ovule primordia during stage 9 and 10 in subepidermal layers (Fig. 5H and data not shown).

**Figure 6:**
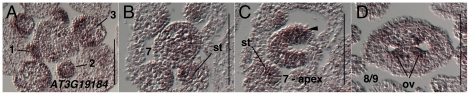
Results of *in situ* hybridization with *AT3G19184* (*REM1*) antisense probe. Numbers indicate floral stages. All panels show transverse (cross sectional) tissue orientation. Arrowheads indicate medial domain expression. Section in panel B is located 8 microns below section in panel C. ov - ovule; st- stamen primordia. Scale bars in all panels are 100 microns. All panels are Col-0 wild type tissue.

The *AT3G19184* (*REM1*) transcript is detected throughout the inflorescence meristem and throughout stage 1–4 floral meristems (Fig. 6A). During stage 5 expression of *AT3G19184* (*REM1*) is strongest in stamen and petal primordia as they arise (data not shown). Expression is detected throughout stage 6 and 7 gynoecia (Fig. 6B). It is strongly detected at the apical regions of stage 7 gynoecia, particularly in medial positions (Fig. 6C). Expression in stage 8 gynoecia is strongest in ovule primordia as they arise. Expression in stage 7 stamen primordia is detected strongly in the precursors of the archesporial and tapetal cells (Fig. 6B, C) and is later expressed in microspores and tapetal cells during stage 9 (data not shown).

**Figure 7:**
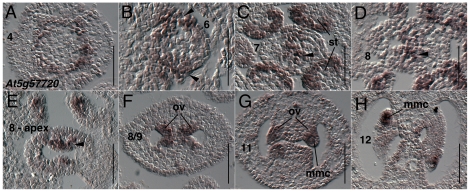
Results of *in situ* hybridization with *AT5G57720* (*REM15*) antisense probe. Numbers indicate floral stages. All panels show transverse (cross sectional) tissue orientation. Arrowheads indicate medial domain expression. ov - ovule; st- stamen primordia; mmc - megaspore mother cell. Scale bars in all panels are 50 microns. All panels are Col-0 wild type tissue.

Expression of *AT5G57720* (*REM15*) in stamen and carpel primordia was previously reported [43]. We first detect expression of *AT5G57720* during early stage 4 as a ring of expression that appears to mark whorl three positions just interior or adaxial to the sepal primordia (Fig. 7A). During stage 6 *AT5G57720* (*REM15*) is detected in the gynoecium in the medial domain, most strongly in abaxial positions (Fig. 7B). During stage 7 *AT5G57720* (*REM15*) is detected throughout the medial domain of the gynoecium (Fig. 7C) and continues to be detected in adaxial portions of the medial domain during stage 8 (Fig. 7 D and E). *AT5G57720* (*REM15*) is detected in ovule primordia as they arise during stage 8 (Fig. 7F) and continues to be expressed in the megaspore mother cell and in nucellar portions of the ovule through stage 12 (Fig. 7G and H). *AT5G57720* (*REM15*) is also detected strongly in stamen tapetal cells during stage 9 (data not shown).

### Additional Approach II candidate genes are preferentially expressed within the medial gynoecial domain

**Figure 8:**
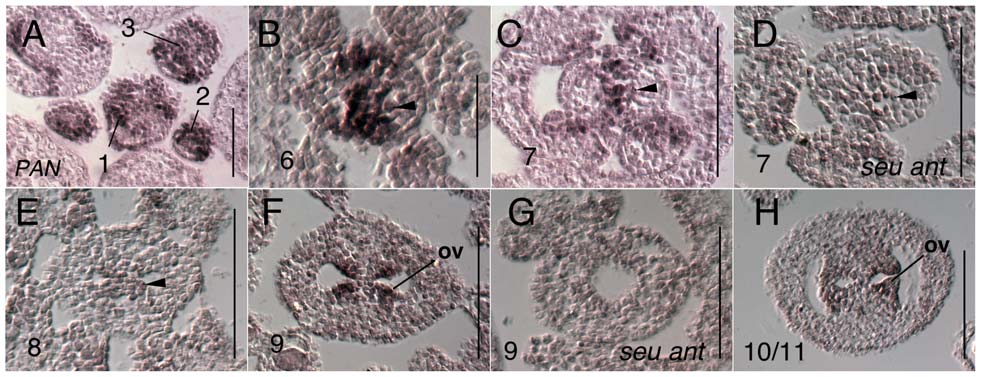
Results of *in situ* hybridization with *PAN* antisense probe. Numbers indicate floral stages. All panels show transverse (cross sectional) tissue orientation. Arrowheads indicate medial domain expression. ov - ovule; Scale bars in all panels are 100 microns, except for panel B - scale bar is 50 microns. All panels are Col-0 wild type tissue except as otherwise noted.

*PERIANTHIA (PAN)* expression patterns have been previously published [44], [55]. *PAN* is detected strongly in the stage 6 gynoecial primordium within the medial domain (Fig. 8B). *PAN* expression continues to be expressed at declining levels within the medial domain throughout stage 7 and 8 (Fig. 8C, and E). Expression is again strongly detected at late stage 8 or early stage 9 in the early ovule primordia (Fig 8F). Expression of *PAN* in the *seu ant* double mutant appeared reduced within the stage 7 medial domain and later (Fig 8D). Additionally *PAN* was not detected in the *seu ant* stage 9 gynoecia (Fig 8G).

**Figure 9:**
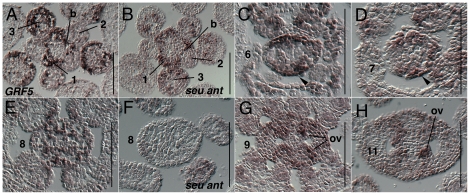
Results of *in situ* hybridization with *AtGRF5* (*AT3G13960*) antisense probe. Numbers indicate floral stages. All panels show transverse (cross sectional) tissue orientation. Arrowheads indicate medial domain expression. ov - ovule; b - boundary region between floral meristem and inflorescence meristem. Scale bars in all panels are 100 microns. All panels are Col-0 wild type tissue except as otherwise noted.

Expression of *AtGRF5* (*AT3G13960*) has been previously reported as strongly expressed in actively growing tissues but only weakly detected in mature tissues suggesting a role in regulation of cellular proliferation [53]. Analysis of a *GRF5:GUS* reporter line revealed expression within the proximal half of the young leaf primordia, a domain with a high proportion of actively dividing cells, however a detailed description of the expression within the flower was not reported [54]. We detected expression of *AtGRF5* (*AT3G13960*) in stage 1 floral primordia, and in a line that marks the boundary between later stage floral primordia and the inflorescence meristem (Fig. 9A). Expression was not detected in floral stage 2 meristems, nor in the inflorescence meristem. Expression was again detected in stage 3 floral meristems in the sepal primordia and then in stamen and petal primordia as they arise during stage 5 (data not shown). Within the gynoecium expression is detected during stage 6 and 7 in the marginal portion of the gynoecium, most strongly detected in abaxial portions of the margin (Fig. 9C and D). During stage 8 *AtGRF5* is detected in a somewhat punctate pattern throughout the gynoecial primordia, but with highest expression within the medial portions (Fig. 9E). *GRF5* is also detected in the ovule primordia are they arise (Fig. 9G) and in subepidermal layers through at least stage 11 (Fig. 9H). Expression of *AtGRF5* in the *seu ant* double mutant tissue appeared to be slightly reduced in stage 1 and stage 3 floral meristems (Fig. 9B) and then strongly reduced within the later stage gynoecia (Fig. 9F)

**Figure 10:**
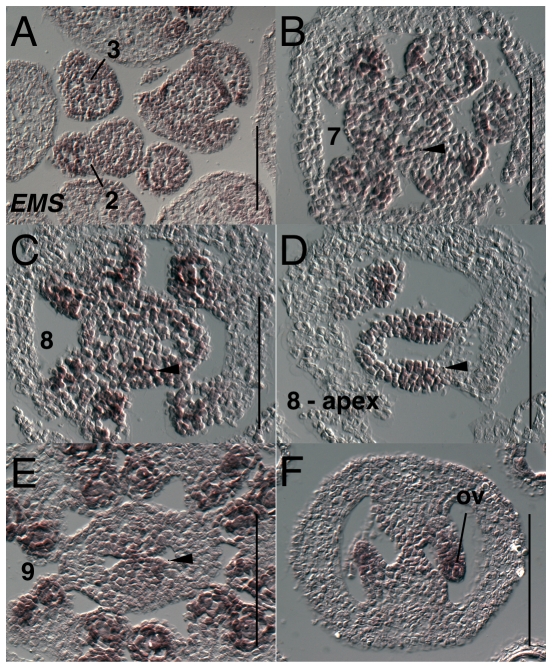
Results of *in situ* hybridization with *EMS* antisense probe. Numbers indicate floral stages. All panels show transverse (cross sectional) tissue orientation. ov - ovule; Scale bars in all panels are 100 microns. All panels are Col-0 wild type tissue.

The expression of *EXCESS MICROSPOROCYTES* (*EMS*) (also named *EXTRA SPOROGENOUS CELLS*) has been previously reported during stamen development [56], [57]. During stage 6 *EMS* is expressed weakly throughout whorls 3 and 4 (data not shown). During stage 7 and early stage 8 *EMS* is expressed throughout the gynoecium, but expression levels are slightly higher in medial domain particularly in the apical region (Fig. 10B, C, D). During stage 8 and 9, expression is evident in ovule anlagen and primordia as they form (Fig. 10E). Expression continues in the ovule primordia in the nucellar and chalazal domains during megaspore mother cell stage and as integuments arise (Fig. 10F).

**Figure 11:**
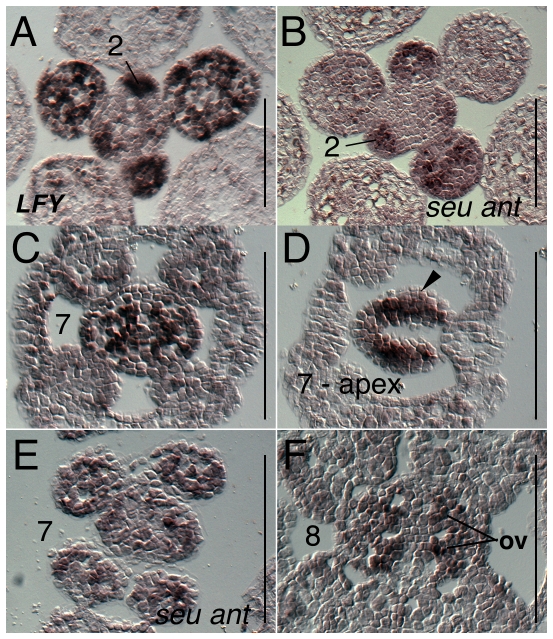
Results of *in situ* hybridization with *LFY* antisense probe. Numbers indicate floral stages. All panels show transverse (cross sectional) tissue orientation. Arrowheads indicate medial domain expression. ov - ovule; Scale bars in all panels are 100 microns. All panels are Col-0 wild type tissue except as marked.

We detected strong *LEAFY* (*LFY)* expression in the stage 1–3 floral primordia as previously reported (Fig. 11A) [58]. In the *seu ant* double mutant tissue expression of *LFY* in the floral stages 1–3 appeared reduced relative to wild type levels (Fig. 11B). During floral stages 4 and 5 *LFY* is expressed strongly in the petal and stamen primordia as they arise, but only weakly detected in the central floral dome (data not shown). During floral stages 6 and 7 *LFY* expression is strongly detected in the adaxial core of the gynoecium and within the medial domain at the apex (Fig. 11C and D). Expression of *LFY* in the stage 6 *seu ant* gynoecia was very reduced relative to wild type levels (Fig. 11E). In early stage 8 wild type tissue *LFY* expression is detectable in the early ovule primordia (Fig. 11 F).

### Other Approach II candidate genes are expressed outside the medial gynoecial domain

Several of the Approach II candidate genes that we assayed by *in situ* hybridization displayed preferential expression within the gynoecial valve domains or expression in both the medial and valve/lateral domains. These data suggest that the effects of the loss of *SEU* and *ANT* on gynoecial development are not specific for the medial domain, but rather alterations of gene regulation occur in both the medial and lateral domains in the *seu ant* double mutants.

**Figure 12:**
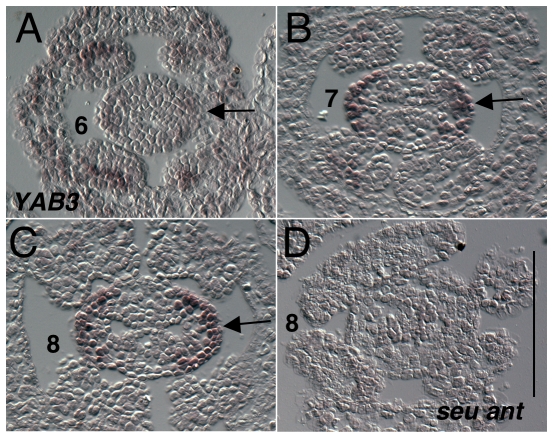
Results of *in situ* hybridization with *YAB3 (At4g00180)* antisense probe. Numbers indicate floral stages. All panels show transverse (cross sectional) tissue orientation. Scale bar in D represents 100 microns for all panels. Arrows indicate abaxial valve domain expression. All panels are Col-0 wild type tissue unless otherwise marked.

*YABBY3* (*YAB3*) (*At4g00180*) expression has been previously reported as expressed within the abaxial portions of all lateral organs derived from both the apical and floral meristems [59]. Expression of *YAB3* is seen in the abaxial valve domains within the gynoecium during stages 6 through 9 (Fig. 12). Expression is fairly weak in the stage 6 gynoecia and becomes stronger in stages 7 and 8 (Fig. 12 A–C). *YAB3* expression in not detected within the medial portions of the gynoecium. Expression in the stage 8 *seu ant* double mutant gynoecium is very reduced or undetectable (Fig. 12D).

**Figure 13:**
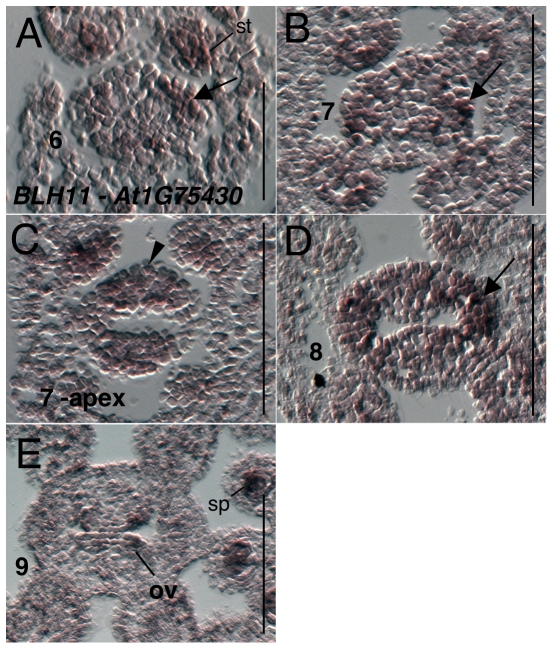
Results of *in situ* hybridization with *BLH11* (*AT1G75430*) antisense probe. Numbers indicate floral stages. All panels show transverse (cross sectional) tissue orientation. Arrowheads indicate medial domain expression. Arrows indicate valve domain expression. ov - ovule; sp - microsporogenic cells. Scale bars in all panels are 100 microns except for panel A where scale bar is 50 microns. All panels are Col-0 wild type tissue.

*BELL-LIKE HOMEODOMAIN 11 (BLH11)* (*AT1G75430)* expression was detected weakly in the inflorescence meristem and stage 1 and 2 floral meristems (data not shown). Expression is more strongly detected in sepal primordia during stages 3 and 4 and in stamen and petal primordia during stage 5 (data not shown). Within the gynoecium expression is detected at stage 6 throughout the primordium, but at higher levels in the valve domains. Expression during stage 7 and 8 is predominantly within the valve domains, but is detected within both valve and medial domains at the gynoecial apex (Fig. 13B, C, and D). Expression is detected in young ovule primordia during stage 8 and 9 (Fig. 13E.) and continues to be detected in nucellar portions of the ovule through stage 11 (data not shown).

**Figure 14:**
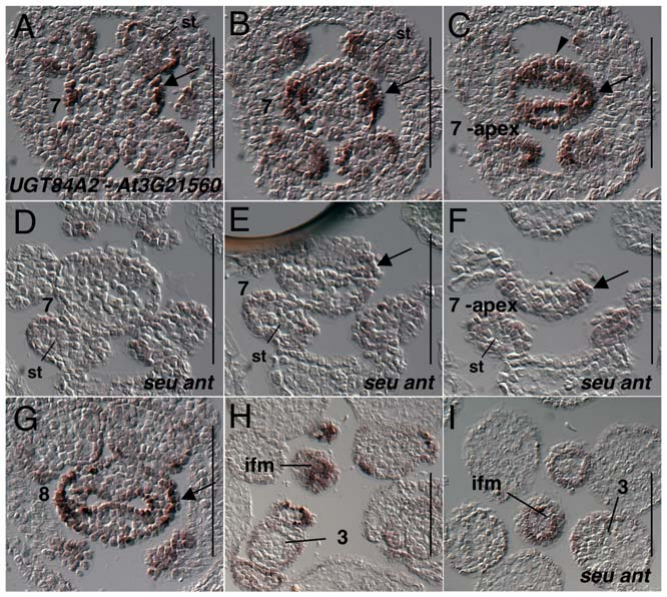
Results of *in situ* hybridization with *UGT84A2 (At3G21560)* antisense probe. Panels A, B, and C as well as panels D, E and F are consecutive serial sections. The section in panel A was located 16 microns basal to the section in panel C and section in panel D was 16 microns basal to the section in panel F. Numbers indicate floral stages. All panels show transverse (cross sectional) tissue orientation. Scale bars are 100 microns in all panels. Arrowheads indicate medial domain expression. Arrows indicate valve domain expression. All panels are Col-0 wild type tissue unless otherwise marked. ifm - inflorescence meristem.

*UGT84A2* (*At3G21560)* is expressed within the inflorescence meristem and stage 1 and 2 floral meristems, chiefly in the L1 cell layer (Fig. 14H). In stage 3 floral meristems *UGT84A2* is strongly expressed in the L1 epidermal cells of the sepals (Fig. 14H). Within the stage 7 gynoecia UGT84A2 expression is detected most strongly in the abaxial portions of the valve domains (Fig. 14A and B). However at the apex of the gynoecium expression is detected in the L1 layer in both medial and lateral/valve domains (Fig. 14 C). During stage 8 expression was detected in the L1 epidermis of the valve domains in both abaxial and adaxial positions (Fig. 14G). Expression in the *seu ant* double mutant tissue was slightly reduced in the inflorescence meristem and young floral buds and strongly reduced in the stage 7 and 8 gynoecia (Fig. 14D, E, F, I).

**Figure 15:**
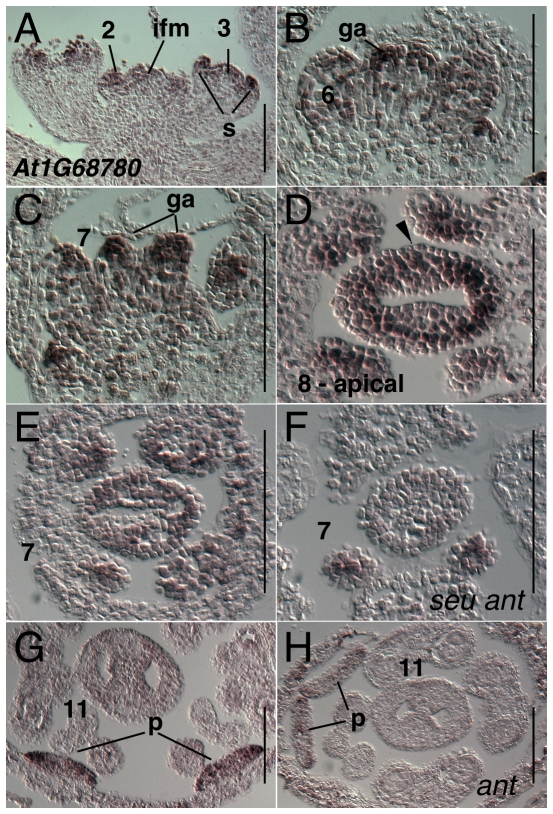
Results of *in situ* hybridization with *At1G68780* antisense probe. Panels A–C show longitudinal tissue sections; panels D-H show transverse (cross) sections. Numbers indicate floral stages. Arrowhead indicates medial domain expression. s - sepal primordia; p - petal primordia; ga - gynoecial apex; ifm - inflorescence meristem. Scale bars in all panels are 100 microns. All panels are Col-0 wild type except as noted.

The gene *At1G68780* is annotated as a member of the RNase inhibitor-like superfamily containing multiple leucine rich repeat InterPro domains (InterPro:IPR001611) [60]. Expression of *At1G68780* was detected weakly throughout the inflorescence meristem and floral stages 1–2 (Fig. 15A). During floral stage 3 expression was strongly detected within the sepal primordia. During floral stages 6 through 8, *At1G68780* is most strongly detected in apical portions of the gynoecium throughout both medial and lateral domains (Figs. 15B through 15D). Gynoecial expression was significantly reduced in the stage 7 *seu ant* double mutant gynoecia. Expression was detected in wild type flowers throughout petal development during floral stages 5 through 12 (Figs. 15B, C, G and data not shown). Expression within the petals was reduced in the *ant* single mutant relative to wild type at stage 11 (Fig. 15H).

## Discussion

Here we report the transcriptomic signature of the *seu ant* double mutant gynoecium relative to wild type and single mutant gynoecia in an effort to characterize both the set of genes important for CMM development and those that are synergistically regulated by the coordinated activities of the *SEU* and *ANT* transcriptional regulators. We have identified a diverse set of transcripts displaying altered expression levels in the *seu ant* double mutant tissues. The analysis of the set of genes displaying reduced accumulation in the *seu ant* double mutant tissue indicates a preponderance of transcriptional regulators including multiple members of the *REPRODUCTIVE MERISTEMS* (*REM)* and *GROWTH-REGULATING FACTOR* (*AtGRF)* families. Our *in situ* hybridization analyses indicate that many of these genes are preferentially expressed within the medial domain of the wild type gynoecia further suggesting a role for these genes during CMM development.

### *GROWTH-REGULATING FACTOR (AtGRF)* family

Members of the *AtGRF* gene family encode proteins with a conserved QLQ domain that functions as a protein/protein interaction domain and a conserved WRC domain that functions as a nuclear localization signal and contains a putative DNA binding C_3_H motif. *AtGRF1*, *AtGRF2*, *AtGRF3* and *AtGRF5* have been shown to regulate growth and development of leaves, cotyledons and floral organs [52], [53], [54]. Over-expression of members of this gene family result in wider leaves and petals while *AtGRF5* single mutants and *AtGRF*1,2,3 triple mutants display narrower leaves and petals. The phenotypic effects of the loss of function of *AtGRF1,2,3* and *AtGRF5* are enhanced by mutations in *GRF-INTERACTING FACTOR1* (*AtGIF1*) [52], [54]. *AtGIF1* encodes a transcriptional co-regulator that physically interacts with members of the *AtGRF* family. The *AtGIF* and *AtGRF* family members are thus likely to support cell proliferation required for the lateral (laminal) expansion of the leaf blade. Additionally, mutations in *AtGIF1* reduce female fertility and this effect was enhanced as the dosage of wild type *GRF* family members was reduced in the *gif1* mutant background [52]. Recently, it has been observed that an *AtGIF* triple mutant, *gif1 gif2 gif3*, develops unfused gynoecia, that lack replum and septal tissues, and contain fewer ovules ([61]; personal communication J. H. Kim) These results support a role for *AtGIF1* and *AtGRF* family members in female reproductive development.

### *REPRODUCTIVE MERISTEMS (REM)* family

A function has been determined for two of the *REM* family transcriptional regulators. *VERDANDI* (*VDD)* is required for female gametophyte development while *VERNALIZATION1* (*VRN1)* is required for the maintenance of the vernalization response [41], [62]. Although *VDD* is expressed within the medial gynoecial domain and was identified in our transcriptomics analysis as a potential regulator of CMM development, *VDD-RNAi* constructs do not disrupt CMM development [41]. Mutations in *At4G31610* (*REM34* - previously *AtREM1*) and *At3G17010* (*REM22*) do not condition obvious developmental defects [42], [63]. Our unpublished analysis of loss-of-function alleles of *At3G53310* (*REM16*) and *At3G19184* (*REM1*) also failed to detect developmental defects. Given the strong expression of several members of the *REM* family during early CMM development, it is possible that *REM* family members share a redundant function that may be revealed in the analysis of higher order mutant combinations.

### Characterization of the transcriptional hierarchies required for CMM development

Although *in situ* hybridization is only a semi-quantitative technique, in most cases the reductions in gene expression in the single and double mutants that were detected in the ATH1 microarray and qRT PCR analyses were confirmed in our *in situ* hybridization experiments. The *in situ* hybridization technique is advantageous in that it allows a finer spatial and temporal characterization of the expression differences between the genotypes. In many cases, our *in situ* hybridization experiments revealed a reduction in gene expression of a candidate gene before an alteration in gynoecial or ovule morphology was apparent in the mutant tissue. In these cases it is unlikely that the reduction of transcript level is simply due to a loss of the tissue in the mutant. However, the data we present here cannot distinguish between direct and indirect transcriptional targets of *SEU* and *ANT* regulation and thus our ability to define the transcriptional hierarchy of CMM development is limited at this time. Future analyses aimed to identify direct transcriptional targets of *SEU* and *ANT* regulation through chromatin-immunoprecipitation or glucocorticoid-inducible activities will help to identify the subset of candidates listed here that are directly regulated by *SEU* and *ANT*. These analyses will help to better delineate the levels of the transcriptional hierarchy required for CMM development and may illuminate the mechanistic basis for the synergistic genetic interaction between *seu* and *ant* mutants during CMM development. Synergistic genetic interactions are commonly observed in animal, plant and fungal systems and yet the mechanistic basis for the synergistic effect typically is poorly understood.

### *SEUSS* may mediate the action of MADS domain-containing protein complexes required for medial domain development

Interestingly, ten of the 31 Approach II candidate genes (including five of the seven *B3* candidate genes) have been previously identified by Gomez-Mena and colleagues as induced in response to the MADS domain-containing transcription factor *AG* [47]. Based on our ATH1 data and follow-up qRT-PCR (Table 3), the levels of *AG* transcript accumulation are not statistically different between the wild-type, *seu*, *ant,* or *seu ant* double mutant in the gynoecial samples. These data suggest that *SEU* and *ANT* do not alter the levels of *AG* accumulation in the CMM, but rather they may work in parallel to *AG* and/or might alter the ability of the AG protein to function. The *SEU* transcriptional adaptor is known to physically interact with dimers of MADS domain DNA-binding proteins (including AP1, SEP3, SVP, and AGL24) during the specification of floral organ identity [21], [29], [30], [31], [64]. We speculate that SEU may function in the developing gynoecium by mediating the action of MADS domain proteins (AG and others) and thus support the expression of a subset of the identified Approach II candidate genes. It is notable that both *VDD* and *REM16* (*AT3G53310*) are direct targets of the MADS protein SEEDSTICK (STK) [41].

### The medial apex of the gynoecium as a developmental domain

Our *in situ* analyses together with the work of other groups indicates that at least 16 of the 31 Approach II candidate genes are expressed preferentially within the medial gynoecial domain with respect to their gynoecial expression. The exact timing and position of expression within the medial domain varies between the candidates. Yet many of these genes similarly display strong expression within the apical-most portion of the medial gynoecial domain. These expression patterns suggest that the medial apex might be functionally distinct from other portions of the gynoecium as early as stage 6. The common expression pattern of many of these candidates suggests that gene regulation events within the apical medial domain of the gynoecium may be critical for the subsequent initiation of ovule primordia from the medial ridge tissues. In this scenario the maintenance of a particular transcriptional or cellular state within the medial apex would be required to maintain the meristematic potential of the medial domain during elongation of the gyneocial tube.

The medial apex of the stage 6 gynoecium is also marked by the expression of *TRYPTOPHAN AMINOTRANSFERASE OF ARABIDOPSIS1* (*TAA1*) [15], [45]. *TAA1* encodes a tryptophan aminotransferase required for the synthesis of auxin via the indole-3-pyruvic acid (IPA) branch of the auxin biosynthesis pathway [45], [65]. We previously demonstrated an enhanced sensitivity of the medial domain to the action of auxin transport inhibitors and suggested a model in which patterning along the medial-lateral axis of the gynoecium requires an auxin dependant signal [15]. Among the list of Approach II candidates, *TAA1* as well as *INDOLE-3-ACETIC ACID INDUCIBLE 18* (*IAA18*), *PINFORMED 1* (*PIN1*) and *LIKE AUXIN RESISTANT2* (*LAX2*) all are known to encode auxin synthesis, transport or response functions [66]. Additional experiments will be required to test the role of these genes during medial domain development.

### Non-cell autonomous functions during CMM development

Several of the Approach II candidate genes displaying reduced expression in the *seu ant* double mutant were not expressed specifically in the medial domain in wild type gynoecia, but rather displayed strong expression in valve domains (e.g. *YAB3, BLH11* and *UGT84A2*). *YABBY* family members are expressed in abaxial portions of aerial lateral organs and support laminal expansion in response to the juxtaposition of abaxial and adaxial fates during organ growth [67]. The loss of CMM development in the *ant yab1 yab3* triple mutant indicates a role for *YABBY* genes during CMM development [18]. The *YABBY* genes likely exert a non-cell-autonomous effect on CMM development suggesting that interactions between the developing valve and medial domains may be important during early gynoecium development. Our transcriptomics data indicates that expression of *YABBY1/FILAMENTOUS FLOWER* (*AT2G45190*) is also significantly reduced (to 45% of wild type levels) in the *seu ant* double mutant (Table S9). However, *YABBY1/FILAMENTOUS FLOWER* did not make our list of Approach II candidates because it did not display a reduction in either of the single mutants.

The alterations of gene expression in the lateral domain of the *seu ant* double mutant point to a role for *SEU* and *ANT* function within the lateral domain. Although the most dramatic gynoecial defects in the *seu ant* double mutant are observed within the CMM and its derived structures, the size of the carpel valve, the overall floral size and plant height are also reduced in the *seu ant* double mutant indicating that the loss of *SEU* and *ANT* activity alters more than just medial domain development [12]. The enhanced effect of the *seu ant* double mutant on CMM development may reflect an enhanced sensitivity of the medial domain to the loss of *SEU* and *ANT* activities.

## Methods

### Transcriptomics data analysis

Whole inflorescences were fixed in ice cold 100% ethanol overnight and then stored for up to one week in 100% ethanol before hand-dissection of gynoecia from floral stages 8–10 under a dissecting scope. RNA was isolated from staged gynoecia using the RNeasy Plant Mini Kit from Qiagen. Linear amplification, labeling, and fragmenting of the cDNA was carried out according to GeneChip 3′ IVT Express Kit instructions from Affymetrix. The initial 25 ng of total RNA was amplified to approximately 11 to 15 micrograms of fragmented and labeled aRNA. Affymetrix ATH1 microarrays were hybridized by Expression Analysis (Durham, NC). Probe intensity data was imported into JMP Genomics 4.1 (SAS, Cary, NC). The CEL or intensity files for each array were compared with a distribution analysis for similarity of the arrays. After visual inspection, none of our arrays was excluded. The data for the arrays was normalized using the Loess Model of Normalization. The probe set values were then summarized by calculation of the mean for each probe set.

Two analyses were run on our data. The first was a simple 1-way ANOVA by genotype. This method identified genes with expression levels that were statically different from that of the mean of the expression values. Class variables were specified as the genotype and the genotype was modeled as a fixed effect. The data was not compared to any baseline, but the LSMeans were run for simple differences of genotype using pFDR for the multiple testing method with an alpha of 0.05. Additionally we required that log2 of the magnitude of the expression level difference between the compared genotype means was greater than 0.35. The fixation method for the data points with large residuals was set as the False Positive Rate and the LSMeans standardization rate was set for Standard Deviation. The second analysis method (Approach II) was directed at detecting genes whose expression was synergistically affected in the *seu ant* double. The null hypothesis tested here was that the value for the *seu ant* double was equal to that of the addition of the *seu* value with the *ant* value. We then accepted all values for the *seu ant* double that were statistically different from the additive estimate. This analysis was done in JMP Genomics using the estimate builder feature. Results of the ANOVA and the calculated statistical significance of non-additivity estimates from the estimate builder function for all 22,810 probe sets are reported in Table S14. We then applied a second criterion to this list by requiring that the mean expression in the *seu* or *ant* single mutant was significantly lower than the wild type mean expression level (by ANOVA). This reduced the list of Approach II candidates to 31 genes (See Table 1). Gene lists were moved to virtual plant [68] to convert Affymetrix probe set IDs to AT gene identifiers and to generate intersection and union sets. GO TERM enrichment analysis was carried out using ChipEnrich [69]. Chip Enrich selects for p<0.001 hypergeometric probability without correcting for multiple testing. The ChipEnrich program also returned statistically overrepresented DNA binding motifs in a 1 kilobase region 5′ to the annotated ATG of the genes in the set and overrepresented transcription factor gene families. ATH1 data sets have been submitted to the Gene Expression Omnibus (GEO) database [70] (series record GSE30492) and the Array Express database [http://www.ebi.ac.uk/arrayexpress/] with experiment number (E-MEXP-3293).

### Quantitative Real time RT PCR and in situ hybridization analysis of candidate gene expression

For analysis of transcript abundances, RNA from stage 8–10 gynoecia isolated for microarray analysis (pre-amplification) was used. cDNA synthesis and qRT-PCR were performed as previously described [12], except we used the SuperScript III First-Strand Synthesis System (Invitrogen) to generate cDNA and the cDNA was diluted 1:4 for qRT-PCR analysis. A single qRT-PCR experiment assayed four biological replicates each of wild type, *seu*, *ant*, and *seu ant* genotypes. Each biological replicate was assayed in triplicate. Results in Tables 2 and 3 are the mean expression of the indicated gene normalized to the expression level of *ADENOSINE PHOSPHORIBOSYL TRANSFERASE1* (*APT1*, At1g27450). Results shown are the average expression normalized to *APT1* and the standard error of the mean for four biological replicates. *APT1* was shown to be unaffected by genotype in our gynoecial RNA samples by comparison of *APT1* expression levels with two other standards (*TUB6*; *AT5G12250* and *G6PD3*; *AT1G24280*) across the four genotypes. Statistical analysis of one way ANOVA was conducted in JMP8 (SAS Institute Incorporated, Cary NC.) using a Tukey-Kramer HSD test and a p value cutoff of 0.05. Sequences of the oligonucleotides used for qRT-PCR analysis are described in Table S13. The *in situ* hybridizations were carried out as reported previously [21] with the following modifications: acetic anhydride and RNase treatment steps were omitted. A detailed protocol is available at http://www4.ncsu.edu/~rgfranks/research/protocols.html.

## Supporting Information

Table S1 Genes under-expressed (reduced accumulation) in the *seu* single mutant via one way ANOVA.
(XLS)

Table S2 Genes over-expressed (increased accumulation) in the seu single mutant via one way ANOVA.
(XLS)

Table S3 Genes under-expressed (reduced accumulation) in the *ant* single mutant via one way ANOVA.
(XLS)

Table S4 Genes over-expressed (increased accumulation) in the *ant* single mutant via one way ANOVA.
(XLS)

Table S5 Over-represented GO categories for gene set displaying reduced expressed in the *seu* single mutant.
(XLS)

Table S6 Over-represented GO categories for gene set over expressed in the *seu* single mutant.
(XLS)

Table S7 Over-represented GO categories for gene set displaying reduced expressed in the *ant* single mutant.
(XLS)

Table S8 Over-represented GO categories for gene set over expressed in the *ant* single mutant.
(XLS)

Table S9 Genes under-expressed (reduced accumulation) in the *seu ant* double mutant via one way ANOVA.
(XLS)

Table S10 Genes over-expressed in the *seu ant* double mutant via one way ANOVA.
(XLS)

Table S11 Over-represented GO categories for gene set displaying reduced expressed in the *seu ant* double mutant.
(XLS)

Table S12 Over-represented GO categories for gene set over expressed in the *seu ant* double mutant.
(XLS)

Table S13 Sequences of oligonucleotides used for qRT PCR analysis of candidate gene expression.
(XLS)

Table S14 Output from JMP Genomics (SAS) ANOVA analysis with values for estimate builder (non-additivity) model for double mutant.
(XLS)
